# Atmospheric Pressure over the Alveolar Ridge

**Published:** 1869-07

**Authors:** W. George Beers

**Affiliations:** Montreal.


### ARTICLE X.

### *Atmospheric Pressure Over the Alveolar Ridge.*

By W. George Beers, Montreal.

In spite of the most perfect impressions and every possible precaution, it is not uncommon to meet with rubber upper sets which drop down at the front of the mouth, in ordinary conversation. No size of palatine air chambers seem to obviate the difficulty, and if adhesion is obtained at all, it is after considerable time, *and a perseverance* on the part of the wearer, which the majority of patients do not possess. We have had our share of trouble with these cases, and particularly of late. One case was that of a lady whose alveoli had absorbed with but little accompanying absorption of the gums. The latter remained soft, though healthy to all appearances, and two years after the insertion of the set of teeth there was no perceptible change. The set gave way in front, and dropped. The other case was that of lady wearing an upper set made by a confrere, which he had perseveringly renewed three times in hopes of securing adhesion, but to no avail. The gums in this instance were hard, and the alveolar process rather more absorbed than usual—the front part being less than the sixteenth of an inch above the level of the palatine bones. We take these two cases as extremes of a condition of gums and alveoli, to which artificial sets are difficult to adapt.

In both these cases, as in all others which we afterwards tried, the sets were wholly completed; and the following remedy was only used and is only recommended as a *dernier resort* in cases where sets drop.

In addition to the palatine air-chamber, we cut four separate round vacuums in the rubber immediately over the part which touched the alveolar ridge when the set was in mouth. Commenced at the second bicuspids on both sides, and ended back of the centrals: the holes a little more than the ordinary depth of an air-chamber.

The result in every case so far has been almost immediate atmospheric pressure, and adhesion of the sets at the very point where they dropped. When the air was exhausted from the mouth, the suction was almost immediate; and by this means we have succeeded in obtaining perfect suction in several cases which previously were failures. In none of these cases has the mucuous membrane been rendered sore by being drawn into the vacuums, though we make our patients provide against this, by leaving their sets out for awhile, during the night for instance, if the gums are at all tender.

We object to air-chambers in vulcanite sets if they can be dispensed with, but there are difficult cases now and then when even the ordinary palatine chamber is insufficient.

Would some of our friends who meet with difficult cases of the kind, try the means here suggested, and report to the *Journal.* We are aware that vacuums have been made over the alveolar ridge of the inferior maxillary for lower sets, but we never heard outside of our own experience of the application of atmospheric pressure principle to that of the superior maxillary.—*Canada Journal of Dental Science.*

